# Benchmark datasets of representative geothermal reservoir models with pseudo-geophysical exploration and well data

**DOI:** 10.1016/j.dib.2024.110828

**Published:** 2024-08-10

**Authors:** Alexandros Patsoukis Dimou, Anna Suzuki, Yusuke Ohta

**Affiliations:** a Institute of Fluid Science, Tohoku University, 2-1-1 Katahira, Aoba-ku, Sendai, Miyagi, Japan; b School of Physics and Astronomy, University of Edinburgh, Peter Guthrie Tait Road, King's Building, Edinburgh, United Kingdom; c Research Institute for Marine Resources Utilization, Japan Agency for Marine-Earth Science and Technology, 2-15 Natsushima-chō, Yokosuka, Kanagawa, Japan

**Keywords:** Geothermal energy, Reservoir simulation, Hydrothermal system, Numerical simulation

## Abstract

A comprehensive investigation of geothermal reservoirs is essential to optimize geothermal energy production and move toward a more sustainable energy future. Various analysis methods and tools have been developed to estimate reservoir conditions and reservoir structures based on geophysical surveys, well data, and other measurement data. In the case of real field data, the actual subsurface structure is unknown, making it difficult to verify the validity of the methods and tools each develops. This data article classifies Japanese geothermal reservoirs and selects two representative structures, which can be representative models for many geothermal fields. Numerical simulations are used to calculate natural conditions and obtain simulated observation data. This paper outlines the methodology employed to construct the reservoir models and to conduct the reservoir simulation. It also describes the approach used to generate resistivity data. The datasets include important reservoir configuration parameters such as rock type, porosity, permeability, rock density, thermal conductivity, and specific heat. It also includes temperature, pressure, and resistivity maps that represent pseudo-geophysical exploration and well data. This comprehensive data set is a valuable resource for further research and analysis in the field of geothermal energy.

Specifications Table

**Table:** 

Subject	Earth and Planetary sciences, Engineering, Energy
Specific subject area	Geothermal reservoir modelling
Type of data	xlsx (Permeability data, Porosity data, Resistivity data, Geothermal Field structural data) Figure (Permeability map, Porosity map, Resistivity map, Reservoir Grid configuration
Data collection	Lithological data, Porosity, Rock Density, Permeability, Thermal Conductivity: based on bibliography. Pressure and Temperature data: By performing numerical simulations with numerical simulator, TOUGH2 and FIGS3C based on porosity, temperature, thermal conductivity data that are also provided with the dataset. Resistivity data: By performing calculations using the equations provided in section 2.3 and using as inputs the porosity, temperature, permeability data that are also provided with this dataset.
Data source location	• Institution: Institute of Fluid Science, Tohoku University • City/Town/Region: Miyagi, Aoba-ku, Sendai • Country: Japan
Data accessibility	Repository name: Open Science Framework Data identification number: DOI 10.17605/OSF.IO/6EB3N Direct URL to data: https://osf.io/6eb3n/
Related research article	none.

## 1 Value of the Data

• Geothermal reservoirs are classified into two main categories with reference to Japanese geothermal reservoir classification. Performing numerical modeling of two geothermal reservoir models, which are representative of the geological characteristics of each reservoir category, can provide a complete dataset for each category.
• The benchmark datasets, which include reservoir configuration parameters, pseudo-geophysical exploration data and well data, can provide a valuable benchmark for validation of various reservoir analysis methods, including machine learning.
• Multiphysics-aware benchmark datasets can provide geologists, geophysicists, and reservoir engineers with a comprehensive, multidimensional perspective.

## 2 Background

This work aims to broadly categorize geothermal reservoirs and to provide representative benchmark datasets reflecting the characteristics of the reservoirs of each category. For geothermal development, it is necessary to understand target reservoirs based on measurable data, such as geological, geophysical exploration, and well data. The methodologies and tools for estimating reservoir structures are called inverse analysis, and various inverse analysis methods have been developed. Recent advances in machine learning have made it possible to develop inverse analysis methods that combine various types of data with multi-physics phenomena [1]. On the other hand, it is impossible to verify the validity of the developed inverse method by using actual field data because it is impossible to understand all the actual subsurface structures. Therefore, possible benchmark datasets obtained by numerical simulation and mathematical modelling can be strongly useful data to validate the inverse analysis methods. The validation of such methodologies has great potential to improve the accuracy of estimations for reservoir configuration parameters based geophysical exploration/well data. This, in turn, will aid in the effective management of geothermal energy production and help foster a clean and sustainable future.

## 3 Data Description

### 3.1 Overview

**Fig. 1:**
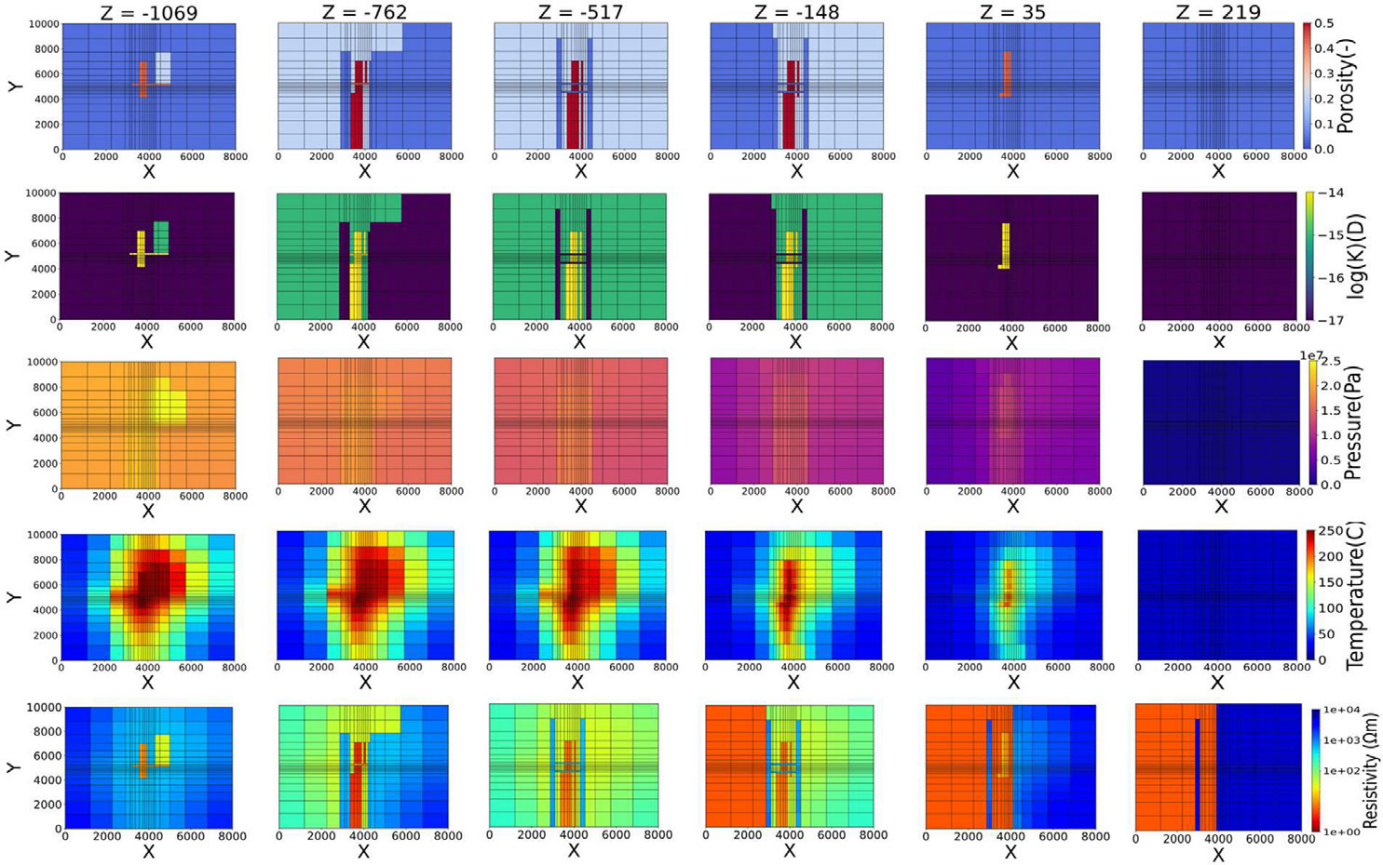
Datasets of main fault-controlled model.

**Fig. 2:**
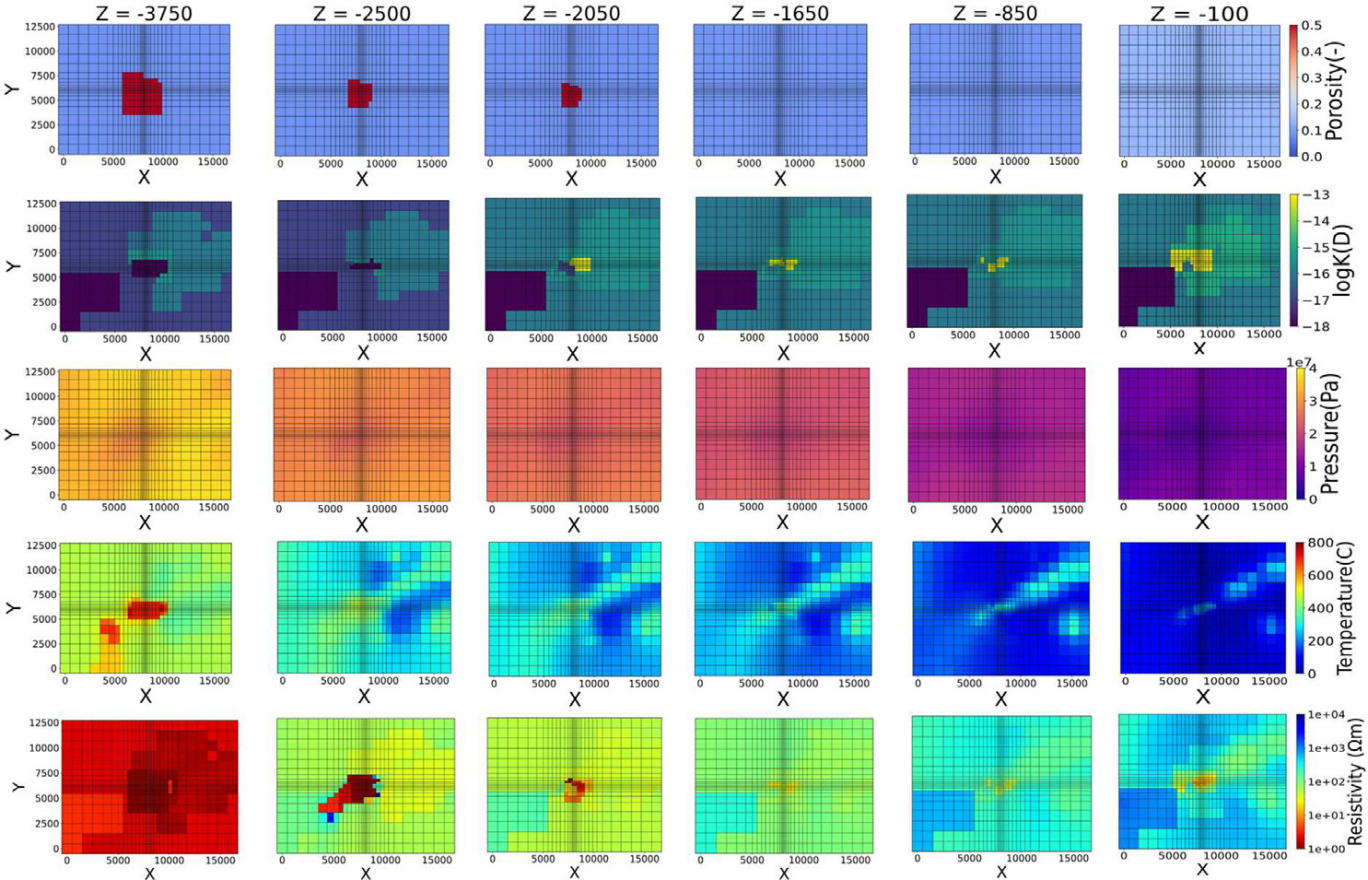
Datasets of rupture zone model.

The datasets include reservoir configuration parameters, pseudo-geophysical exploration data and well data that comprise two representative geothermal reservoir models: “main fault-controlled model” with reference to the Ogiri geothermal field and “rupture zone model” with reference to the Kakkonda geothermal field in Japan. All the data are stored in a data repository [2]. The lists of data for the main fault-controlled and rupture zone models are plotted in Fig. 1, Fig. 2, respectively.

### 3.2 Grid information

**Fig. 3:**
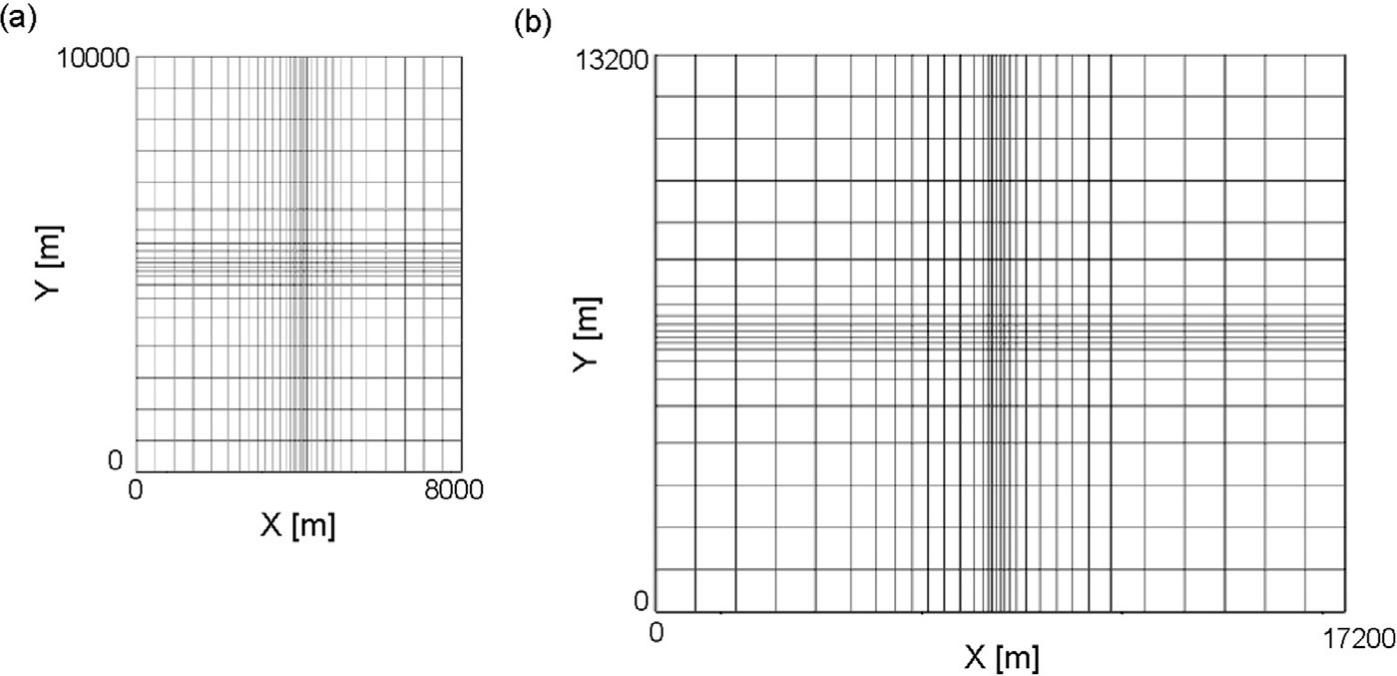
Grid configurations of (a) main fault-controlled model and (b) rupture zone model.

The grid information of the main fault-controlled model and the rupture zone model are stored in files Grid_Data_Main_Fault_Controlled.xlsx and Grid_Data_Rapture_Zone.xlsx in the data repository and shown in Fig. 3. The data can be used for grid construction.

### 3.3 Reservoir configuration parameters

The reservoir configuration parameters for each model are given in the respective grids. The parameters are as follows: (1) rock type, (2) porosity, (3) permeability, (4) rock density, and (5) heat conductivity. All data can be found in the data repository (Input_Data_Main_Fault_Controlled.xlsx, Input_Data_Rapture_Zone.xlsx). The porosity and permeability maps of each model can be seen in Fig. 1, Fig. 2.

### 3.4 Pseudo-geophysical exploration/well data

The resistivity data provided in this work represent geophysical exploration obtained data, and the temperature and the pressure data represent well obtained data. These parameters are given in the respective grid cells. The pressure, temperature, for the two models were obtained by numerical simulation while the resistivity data are calculated using analytical models. The pressure, temperature and resistivity results are available in the data repository (P_T_R_Data_Main_Fault_Controlled.xlsx and P_T_R_ Data_Rapture_Zone.xlsx). Furthermore, the pressure, temperature, and resistivity maps of each model can be seen in Fig. 1, Fig. 2.

## 4 Experimental Design, Materials and Methods

### 4.1 Reservoir classification

Geothermal reservoirs in Japan are separated into three main categories [3]. The categories are reservoirs regulated by main faults (main fault-controlled), reservoirs regulated by numerous fault systems (rupture zone), and reservoirs regulated by other factors such as intrusive rocks (other factors).

The main fault-controlled category includes reservoirs that are distributed along a distinct means layer. Examples of main fault-controlled geothermal reservoirs are reservoirs at Yanaizu-Nishiyama, Otake, Hachobaru, and Ogiri areas. In the case of the Ogiri geothermal field, the strike dip of the main faults, the Ginyu faults, are all in the same direction, and many of them are considered to be high-angle and quite wide.

The rupture zone category includes reservoirs that are distributed in numerous fault zones, for example, geothermal reservoirs located in the Tohoku region such as the Kakkonda shallow reservoir, Sumikawa, Uenotai, and Onuma areas. In the case of the shallow reservoir of Kakkonda geothermal field no major faults are recognized, and the reservoir is dominated by horizontal faults and high-angle vertical fracture zones along the strata boundaries.

**Fig. 4:**
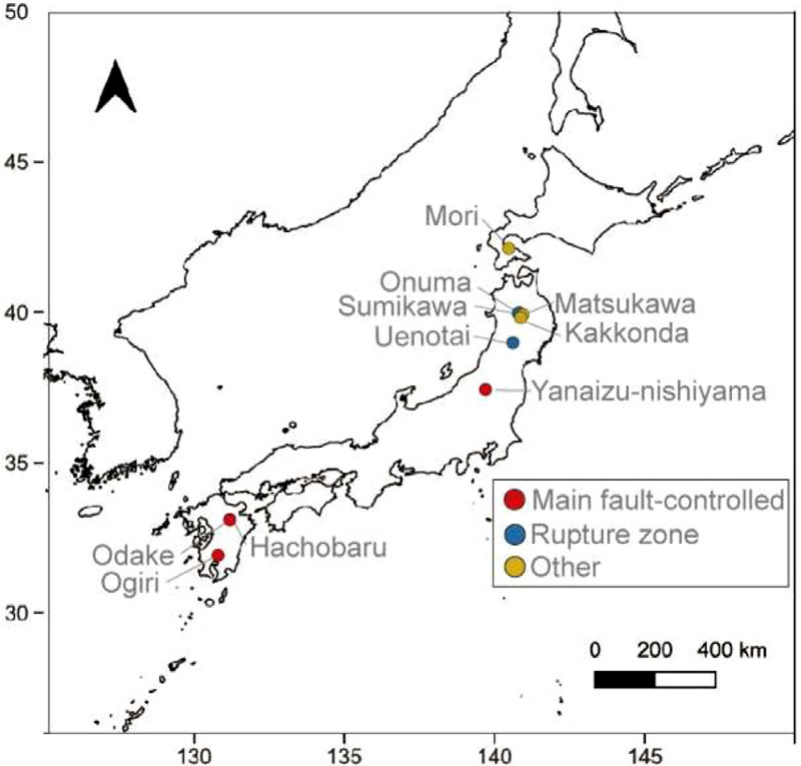
Three categories and locations of geothermal reservoirs. The map uses Natural Earth Data [4].

Examples of other factor geothermal reservoirs are reservoirs dominated by intrusive rock bodies and can be found in Mori, Matsukawa, and Kakkonda areas. In the case of the deeper Kakkonda field, new granite is present as the intrusive rock, and highly permeable fractures are generated in the rock body and at the rock body margins. In the case of the Mori geothermal field, the important fault zones that constitute the geothermal reservoir are mainly developed inside and outside the caldera. The three categories and locations of geothermal reservoirs are shown in Fig. 4.

Since each of the other factor reservoirs is unique, the representative reservoirs were selected from the main fault-controlled reservoir category and the rupture reservoir category. For the main fault-controlled model, the Ogiri field was selected as the representative area because the details of the numerical model were available by Kumamoto et al. [5] and Tateishi et al. [6]. For the rupture zone model, the Kakkonda field was selected as a representative area because a numerical model was available [7].

### 4.2 Reservoir configuration parameter setup

The numerical model representing a main fault-controlled reservoir was constructed with reference to the Ogiri geothermal field, located in Kagoshima prefecture, southern Kyushu, Japan. Since the objective of providing the datasets was not to accurately reproduce the Ogiri geothermal field, but to prepare artificial datasets of a representative main fault-controlled model, any optimization using measured data was not conducted to ensure strict alignment with real-world conditions. A generic geothermal reservoir simulator, TOUGH2 [8] was used for the numerical model construction. Rock types [8], permeability, porosity, and thermal conductivity were based on values from [5,9,10]. Porosity values were taken from Hokkaido Geological Survey Association. Specific heat values were taken from Kitano et al. [11]. Density was obtained from [12] and the Shimanto Group referred to as New Energy and Industrial Technology Development Organization (NEDO) [13]. The computational domain was 8 km × 10 km × 2.3 km and divided into 19 × 20 × 22 grid blocks. Horizontally, the grid was divided to subdivide the reservoir center, and vertically, 22 layers of 61.4 m sized blocks were stacked on top of each other.

The numerical model for the rupture zone model was based on the Kakkonda geothermal field which is located in the northeastern Japan. The model was constructed using FIGS3C (JMC Geothermal Engineering Co., Ltd. and Technical Software & Engineering Inc.) [7]. The horizontal domain is divided into a 32 × 21 grid and the overall size is 17.2 km and 13.2 km in the two axes respectively. Vertically, the study area is divided into 27 layers with different thicknesses, ranging from an elevation of 600 m, slightly below the surface in the Kakkonda area, to an elevation of −4,000 m, sufficiently deeper than the bottom of the well in WD-1a. Rock density, heat capacity, and thermal conductivity are homogeneous, with values of 2500 kg/m^3^, 1000 J/(kg K), and 2.3 W/(m K), respectively. Porosity is homogeneous in each layer and decreases with depth from 18.3 % in the upper layer to 8.5 % in the lower layer. For the permeability, the study area was divided into several regions: shallow reservoirs, deep reservoirs, the margins of the Kakkonda granite, and the northeast area away from the wellbore; while in other studies [7] have considered anisotropy in permeability, in this study, it is assumed that the permeability given to each grid block is constant and independent of direction.

### 4.3 Reservoir simulation

For the calculation of temperature and pressure distribution within the main fault-controlled model, the TOUGH2 software is used [8]. To obtain the pressure and temperature distribution with the reservoir models numerically initial temperature and pressure conditions as well mass and heat flux in and out of the geothermal reservoir is required. The initial conditions for the simulation are a temperature of 15 °C and a pressure of 9.804 × 10^4^ Pa for the top layer. The lateral parts of the simulation model are impermeable to flow and adiabatic to heat. A single cell in the western shallow zone with the coordinates of the center of the grid (*X* = 4350, *Y* = 600, and *Z* = −87.5) is set as an outflow region using a mass rate of 4 kg/s. The mass and heat recharge of the system is specified at the bottom layer with a flow rate of 45 kg/s and specific enthalpy of 1.096 × 10^6^. The numerical simulation is then set to run till steady-state conditions are achieved and the pressure and temperature data provided in the dataset are obtained.

For the rupture zone model, the boundary conditions were set considering the topography of the Kakkonda area [7]. The top boundary is open with respect to mass and heat flow. The temperature at the top layer is 20 °C. The pressure was set considering the elevation of the ground surface at each grid. The temperature at the bottom is set at 480 °C, but some grids are set at 1000 °C, assuming some penetrating rock. The bottom layer is closed to mass flow. Since the origin of the fluid in the deep reservoir seems to be meteoric water, it is likely that there is little ascending flow in the zone of conductive heat transfer. The rock may be ductile, and the fluid may not flow. The numerical simulation is run till steady-state condition is achieved and the pressure and data that are provided with this data set are obtained.

### 4.4 Resistivity calculation

**Fig. 5:**
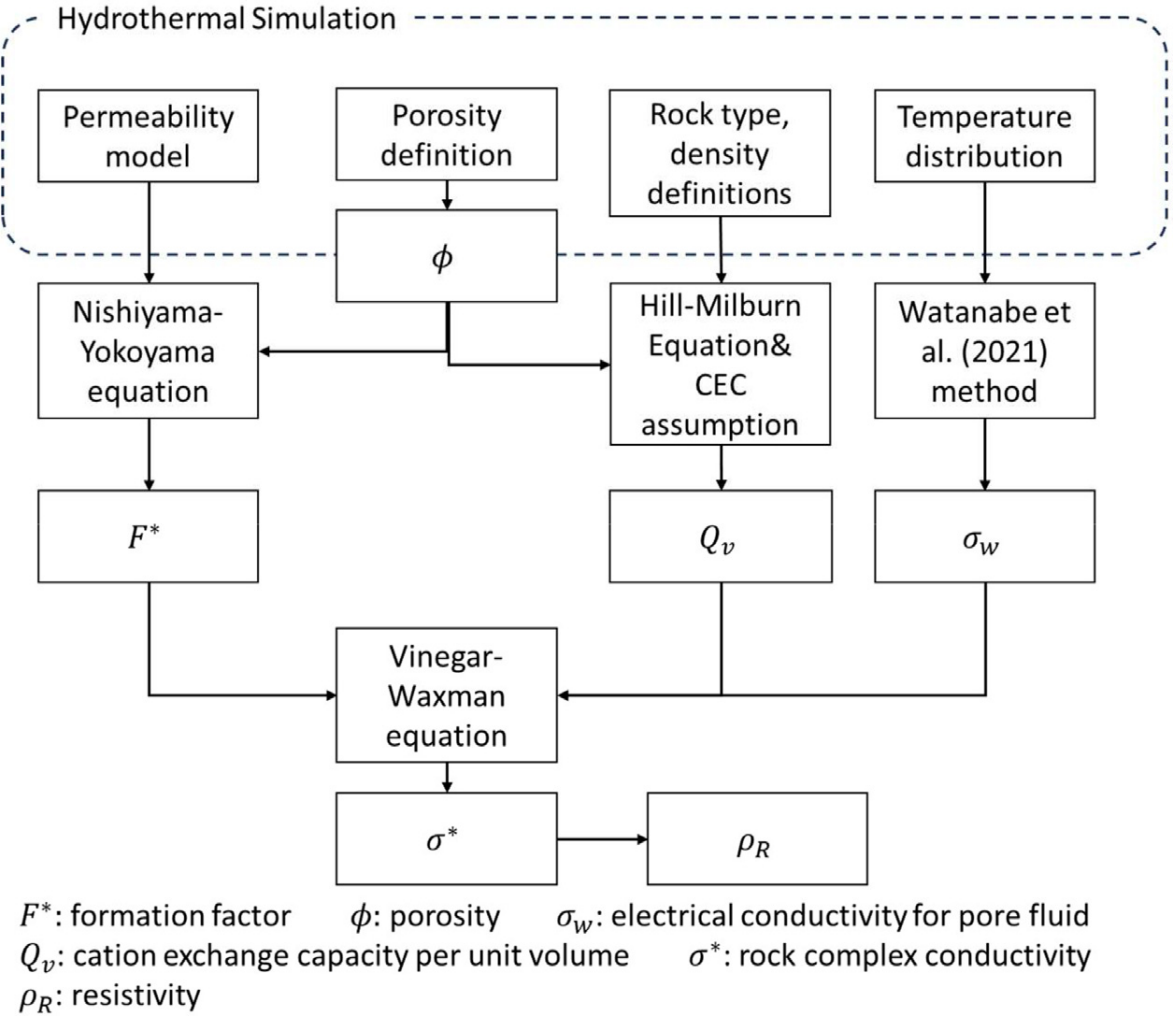
Workflow diagram for resistivity data generation from reservoir simulation.

The resistivity model mirrors the hydrothermal simulation grid. The methodology to generate resistivity data can be seen in Fig. 5. Both the main fault-controlled model and the rupture zone model necessitate adjustments based on their unique geological settings and the influence of diverse physical parameters on rock properties.

The formation factor, $F^{*}$, was derived using the critical pore radius, $r_{cr}$. The equation for $r_{cr}$ proposed by Nishiyama and Yokoyama [14] is,

(1) $$k=8.5{\left(\phi r_{cr}^{2}\right)}^{1.3},$$

(2) $$k=0.014\left(\frac{\sigma_{\theta }}{\sigma_{w}}\right)r_{cr}^{2},$$

Here, $\sigma_{\theta }$ represents the rock's conductivity due to pore fluid conductivity, and $\sigma_{w}$ is the conductivity of the pore water. The above equation is applicable to various rocks, including sandstone and carbonate rock. By incorporating ϕ and k, the $\sigma_{\theta }/\sigma_{w}$ ratio was established. Consequently, the formation factor $F^{*}$ was calculated as $\sigma_{\theta }=\sigma_{w}/F^{*}$, based on Archie's equation. The conductivity of the pore fluid was calculated using the equation from Watanabe et al. [15]. This was based on assumed values for porewater salinity and temperature profiles from hydrothermal simulations. The salinity assumptions varied between the main fault-controlled model and the rupture zone model. For the main fault-controlled model, Cl^−^ ion concentration measurements in the Ogiri area [16] were taken from the circulating reservoir fluid, resulting in an equilibrium concentration of 595 (mg L^−1^). This value was then converted to a NaCl concentration of 0.107 (wt.%), which was consistently used as the representative concentration. For the rupture zone model, the following Cl^−^ concentrations were determined based on depth (*z*): 4.3 × 10^5^ (ppm) [*z* < −3100]; 2.3*z* − 2.8 × 10^3^ (ppm) [−3100 ≤ *z* < −1500]; 0.2 z + 300 (ppm) [−1500 ≤ *z* < 0]; 300 (ppm) [0 ≤ *z*]. These simplified assumptions are based on the salinity of the fluid in the thermal convection and heat transfer zones of the Kakkonda as identified in previous studies [e.g., 17].

Although some grids could not be calculated using the Watanabe formula, the maximum porewater conductivity of 140 (S/m) was consistently applied to these exceptional cells as a representative value. To determine the added conductivity in rocks with clay minerals, the cation exchange capacity per unit volume, $Q_{v}$ (meq cm^−3^) was used. Hill and Milburn [18] proposed the equation:

(3) $$Q_{v}=\frac{CEC\rho_{g}\left(1-\phi \right)}{\phi }.$$

Here, $\rho_{g}$ represents the rock's density (g cm^−3^), and CEC denotes cation exchange capacity per unit weight (meq g^−1^). The prescribed $\rho_{g}$ and ϕ values for each rock type in the hydrothermal simulation were utilized for each calculation. For CEC values, 1 was assigned if the rock type was assumed to be a clay alteration zone, and 0.0005 was used otherwise, based on clay-rich and pure sedimentary rock analyses conducted by Revil et al. [19]. In the rupture zone model, all CEC values were consistently set at 0.0005.

The complex conductivity, denoted as $\sigma^{*}$, was determined using the Vinegar and Waxman equation [20]. The equation is given by:

(4) $$\sigma^{*}=\frac{1}{F^{*}}\left(\sigma_{w}+BQ_{v}\right)+i\left(\frac{1}{F_{q}}\lambda Q_{v}\right)$$

where $F^{*}$ represents the Waxman-Smits Formation Factor, which is treated here as identical to the Formation Factor obtained in former. B is the equivalent ionic conductance of the clay exchange function of $\sigma_{w}$ (mho cm^2^ meq^−1^), which is expressed by the following equation.

(5) $$B=0.0383\left[1-\text{expexp}\;\left(-50\;\sigma_{w}\right)\;\right]$$

The formation factor for quadrature conductivity, $F_{q}$, is defined by $F_{q}=F^{*}\phi$. The parameter λ serves as the proportionality constant for the length of the clay-rich pore zone, under the assumption that the pores within both the clay-rich and clay-poor zones are configured in a series circuit. For this study, λ=0.07 was obtained by averaging the outcomes determined through the application of Eq. (4) to empirical data from geothermal regional rock cores collected in the Kakkonda field. i stands for an imaginary unit. Finally, $\sigma^{*}$ was converted to resistivity $\rho_{R}$ (Ωm), utilizing the equation: $\rho_{R}=1/\left|\sigma^{*}\right|.$

## Limitations

The data were obtained by numerical simulation, which does not take into account influences other than hydrothermal flow, such as chemical and mechanical reactions. Electrical resistivity was calculated indirectly using theoretical equations and reference data from rock sample studies. These are based on several assumptions and physical modeling. Those theories are based on several assumptions. Since it is not a perfect simulation of the field, there are differences from the actual Ogiri and Kakkonnda fields.

## Ethics Statement

The authors dully adhered to ELSEVIER ‘Ethics in publishing’ policy. No ethical issues are associated with this work.

## CRediT authorship contribution statement

**Alexandros Patsoukis Dimou:** Data curation, Visualization, Writing – original draft. **Anna Suzuki:** Conceptualization, Methodology, Investigation, Data curation, Validation, Writing – review & editing. **Yusuke Ohta:** Formal analysis, Data curation, Writing – review & editing.

## Data Availability

Benchmark Datasets of representative geothermal reservoir models (Original data) (Open Science Framework (OSF)).
